# *Fusobacterium nucleatum* Promotes the Development of Ulcerative Colitis by Inducing the Autophagic Cell Death of Intestinal Epithelial

**DOI:** 10.3389/fcimb.2020.594806

**Published:** 2020-11-27

**Authors:** Wenhao Su, Yongyu Chen, Pan Cao, Yan Chen, Yuanmei Guo, Siwei Wang, Weiguo Dong

**Affiliations:** ^1^Department of Gastroenterology, Renmin Hospital of Wuhan University, Wuhan, China; ^2^Key Laboratory of Hubei Province for Digestive System Disease, Renmin Hospital of Wuhan University, Wuhan, China

**Keywords:** *Fusobacterium nucleatum*, ulcerative colitis, autophagy, cell death, chloroquine

## Abstract

There is a growing body of evidence which suggests that intestinal microbiota, especially *Fusobacterium nucleatum* (*F. nucleatum*), are associated with intestinal immune disease such as ulcerative colitis (UC). The mechanism by which *F. nucleatum* promotes intestinal epithelial cell (IEC) death remained undefined. Here, we investigated the potential mechanisms about how *F. nucleatum* aggravates IEC death in UC. We first detected the abundance of *F. nucleatum* in UC tissues and analyzed its relationship with the clinical characteristics of UC. Next, we explored whether *F. nucleatum* promotes intestinal epithelial cell death *in vitro* and *in vivo*. Furthermore, we extracted lipopolysaccharide (LPS) of the *F. nucleatum* and examined whether *F. nucleatum* exacerbates UC *via* LPS. Our results indicated that *F. nucleatum* was abundant in UC tissues and was correlated with clinical characteristics. In addition, we demonstrated that *F. nucleatum* and its LPS aggravated IEC death by promoted IEC autophagy. Furthermore, autophagy inhibitors, chloroquine (CQ), 3-methyladenine (3-MA) or Atg5 silencing prevented IEC death mediated by *F. nucleatum*, which suggests *F. nucleatum* may contribute to UC by activating autophagic cell death. All our results uncover a vital role of *F. nucleatum* in autophagic cell death and UC, giving rise to a new sight for UC therapy by inhibiting excessive IEC autophagy and autophagic cell death.

## Introduction

Ulcerative colitis (UC) is a chronic, idiopathic, relapsing immunologic disorder of the gastrointestinal tract (Park et al., 2017). UC is considered a worldwide healthcare issue, with its steady increase in prevalence (Jeong et al., 2019). In recent years, the incidence and prevalence of UC have a dramatic rise in Asia, which makes it get more attention (Hou et al., 2009). The current treatment strategy of UC is mainly to use anti-inflammatory drugs, immunosuppressive agents, steroids and biological agents to control disease activity. Thus, it is essential to deepen the understanding of the pathogenesis in UC for further treatment.

Intestinal microbes have been taken into account as an important factor in the pathogenesis of UC (Bashir et al., 2016). *Fusobacterium nucleatum* (*F. nucleatum*) is a common colonizer in the oral cavity and intestinal mucosa of human hosts. Studies have found that *F. nucleatum* is closely related to intestinal diseases such as inflammatory bowel disease (IBD) and colorectal cancer (CRC) by promoting intestinal inflammation and the release of inflammatory factors (Kumar et al., 2017; Weng et al., 2019). It has been shown that *F. nucleatum* can aggravate UC by regulating M1 microphage polarization (Liu et al., 2019). However, the mechanism of *F. nucleatum* in the pathogenesis of UC has not been fully elucidated.

Autophagy plays a crucial role in regulating the interaction between gut microbiota and host immunity (Kim et al., 2019). Genetic variations of autophagy genes has been identified to be the pathogenic factors of IBD (Nguyen et al., 2013). Studies has showed a mutual regulation between autophagy and inflammation. Previous studies have showed that loss of functional autophagy promote inflammasome activation and secretion of inflammatory factors such as IL-18 and IL-β, which leads to a higher susceptibility to inflammation (Larabi et al., 2020). However, some studies found that autophagy activation is accompanied with excessive intestinal inflammatory response and epithelial injury in colitis mice (Shen et al., 2018) and high levels of autophagy is a potential signal for cell death (Denton and Kumar, 2019). *F. nucleatum* has been found to activate IEC autophagy pathway, promote cell apoptosis and proinflammatory cytokine production in oral and gut (Dharmani et al., 2011; Yu et al., 2017; Kang et al., 2019). However, the exact role of autophagy in *F. nucleatum*-associated UC is still unclear.

Here we studied whether and how *F. nucleatum* affects IEC function in UC patients. Our results demonstrate that *F. nucleatum* is abundant in UC patients compared with healthy controls. Moreover, enrichment of *F. nucleatum* activates IEC autophagy and exacerbates IEC autophagic death *in vivo* and *in vitro*. Our data provide a potential strategy for UC treatment by inhibiting excessive autophagy flux and epithelial cell death.

## Materials and Methods

### Collection of Clinical Samples

All study samples were obtained from patients between 2018 and 2019 who were treated at Wuhan University People’s Hospital (Hubei, China). This study was approved by the Medical Ethics Committee of Wuhan University People’s Hospital (approval number: 2018K-C089), and written informed consent was obtained from each participant. Endoscopic biopsies were collected from inflamed intestinal mucosal from UC patients. The diagnosis of UC was based on clinical, endoscopic and histological criteria. The clinical data of each patient were collected from the hospital records. Formalin-fixed and paraffin-embedded samples were retrieved from Pathology department archives. The control group was composed of age-matched healthy patients who underwent an endoscopy which resulted in a normal endoscopy and biopsies. Clinical disease activity was assessed using the Colitis Activity Index. Fresh UC tissue specimens were collected from participants of ≥19 years of age with biopsy proven. Patients were screened and those with Crohn’s disease, infectious diarrhea, clinical conditions requiring emergency management, primary sclerosing cholangitis, pregnancy, prednisolone use within the last 2 weeks, biologicals or immunosuppressants within the past 2 years, history of fecal microbiota transplantation (FMT), antibiotic use within previous 3 months and recent malignancy were excluded. Formalin-fixed, paraffin-embedded UC intestinal tissues for FISH analysis were obtained from the pathology department archives between February 2018 and December 2019. Clinicopathologic data for each patient were obtained from the hospital records.

### Bacterial Strains and Cell Lines

The human normal epithelial cell line NCM460 was maintained in Dulbecco’s modified Eagle medium (DMEM) supplemented with 10% fetal bovine serum at 37°C, 5% CO_2_. *F. nucleatum* was incubated for 3 days in fastidious anaerobe broth (FAB) under anaerobic conditions (10% H_2_, 5% CO_2_, and 85% N_2_) at 37°C. *Escherichia coli* strain (Tiangen, China) was cultured in Luria–Bertani (LB) medium for 16 h at 37°C with shaking at 200–220 rpm. The *F. nucleatum* suspension was centrifuged at 2,500×*g* for 5 min and then resuspended in antibiotic-free DMEM before infecting normal epithelial cells.

### DSS-Induced Mouse Model of Colitis

All mice experiments were performed with male mice of 6 to 8 weeks age unless otherwise stated. C57BL/6 mice were given 2 mg/ml streptomycin in drinking water for 3 days to ensure the consistency of common microflora and facilitate colonization of *F. nucleatum*. *F. nucleatum* resuspended in PBS (10^9^ CFU/ml) or PBS alone was administered to mice by gavage every day for 2 weeks. After 2 weeks of infection, mice were administered either normal water or water containing 2.5% (wt/vol) dextran sulfate sodium (DSS) (molecular weight 36 to 50 kDa; MP Biomedicals) for 7 days to induce colitis. To examine the effect of CQ on colitis, mice received *F. nucleatum* gavage or PBS meanwhile injected intraperitoneally with CQ (50 mg/kg of body weight) dissolved in PBS every two days.

### Measurement of Colitis Severity

Mice were observed once daily for weight, body weight, morbidity, stool consistency, and the presence of gross blood in the feces and at the anus. On day 7 after 2.5% DSS treatment, mice were sacrificed, and the colon was quickly isolated for measurement of length. The distal colon was collected and fixed in 10% buffered formalin for 24 h for histological staining (H&E) and fluorescence *in situ* hybridization (FISH). The histological scores were calculated by three pathology staffs. The histological scores were determined blindly as follows: Damaged area: 0: n/a, 1: ≤25%, 2: ≤50%, 3: ≤75%, 4: ≤100%. Muco-depletion of glands: 0 = none, 1 = mild, 2 and 3 = moderate, 4 = severe. Tissue damage: 0= no mucosal damage, 1 = discrete epithelial lesions, 2 = surface mucosal erosion or focal ulceration, 3 = extensive mucosal damage and extension into deeper structures of the bowel wall. Inflammatory cell infiltration: 0 = occasional inflammatory cells in the lamina propria, 1 =increased numbers of inflammatory cells in the lamina propria, 2 = confluence inflammatory cells, extending into the submucosa, 3 = transmural extension of the infiltrate. The total histopathological score was determined by summation of the scores from each category.

### RNA Extraction and Real-Time PCR

Total RNA was extracted using Trizol reagent (Invitrogen, Carlsbad, CA, USA), and 1 μg of total RNA was reverse transcribed using the PrimeScript RT Reagent Kit (Perfect Real Time; Takara, Shiga, Japan). Real-time quantitative PCR was performed in triplicates on an Applied Biosystem7900 quantitative PCR system (Applied Biosystems, Foster City, CA, USA). The Ct values obtained from different samples were compared using the 2^−ΔΔCt^ method. Glyceraldehyde-3-phosphate dehydrogenase (GAPDH) served as the internal reference transcripts. The primers are shown in **Supplementary Table 1**.

### Fluorescence *In Situ* Hybridization

Five-micrometer-thick sections were prepared and hybridized following the manufacturer’s instructions (FOCOFISH, Guangzhou, China). The sequence of the *F. nucleatum*-targeted probe (FUS664; FITC-labeled) was 5’-CTT GTA GTT CCG C(C/T) TAC CTC-3’. Slides were examined using a microscope (BX53F; Olympus, Tokyo, Japan). Five random 200× magnification fields per sample were evaluated by an observer who was blinded to the experimental protocol, and the average number of bacteria per field was calculated. We defined a negative or positive of *F. nucleatum* as an average of < 10 or > 10 visualized FUS664 probes per field, respectively.

### Electron Microscopy

NCM460 cells were treated with PBS, *E.coli* (DH5α) or *F. nucleatum* and fixed with 2.5% glutaraldehyde. Samples were post-fixed with 1% osmium tetroxide, dehydrated in ethanol and propylene oxide, and embedded in epoxy resin. Subsequently, embedded samples were cut into ultra-thin sections (50–70 nm) stained with 3% uranyl acetate and lead citrate. Finally, the sections were observed by JEM-1230 electron microscope (JEOL, Tokyo, Japan).

### Knockdown of Atg5 With shRNA

siRNA targeting the human Atg5 gene (siAtg5) and nontargeting siRNAs (control siRNAs) were purchased from Genechem (Shanghai, China). Cells were cultured and transfected with siRNAs according to the supplier’s instructions. Lipid-based transfections were achieved with Lipofectamine 6000 (Beyotime, China) according to the manufacturer’s protocol. Cells were incubated with the siRNA complex for 72 h, and protein was extracted for assessing transfection efficiency by WB.

### Western Blotting

Total cellular protein was isolated from cultured cells and mice colons with a protein extraction solution (Beyotime, china). Proteins were subjected to 10 and 12% sodium dodecyl sulfate polyacrylamide gel electrophoresis (SDS-PAGE) and transferred to polyvinylidene fluoride (PVDF) membranes at 0.25 A (1 h, 4°C) using a wet-blotting apparatus (Bio-Rad). Membranes were blocked with 5% non-fat milk for 2 h at room temperature, and then incubated overnight at 4°C with the primary antibodies diluted in PBST. Membranes were then incubated with appropriate secondary antibodies for 1 h at room temperature. The protein signals were detected by using the ChemiDocTMXRS+ system (Bio-Rad). For WB, primary antibodies against the following targets were used:LC3 (CST), P62 (CST), Atg5 (CST), Bcl-2 (Proteintech), Bax (Proteintech), and GAPDH (Bioworld).

### Statistical Analyses

Statistical analysis was performed using GraphPad Prism software version 8.0 and SPSS Statistics 20.0 software. Data are expressed as the means ± SDs. Associations between *F. nucleatum* abundance and patient’s clinical characteristics were determined using Pearson’s Chi-square test or Fisher’s exact test. Normally distributed data were analyzed by Student’s t test. Differences among multiple groups were evaluated for significance using one-way ANOVA combined with Bonferroni’s *post hoc* test. Statistical significance was defined as P <0.05.

## Results

### *F. nucleatum* Is Abundant in UC Tissues and Linked to Clinical Characteristics

**Figure 1:**
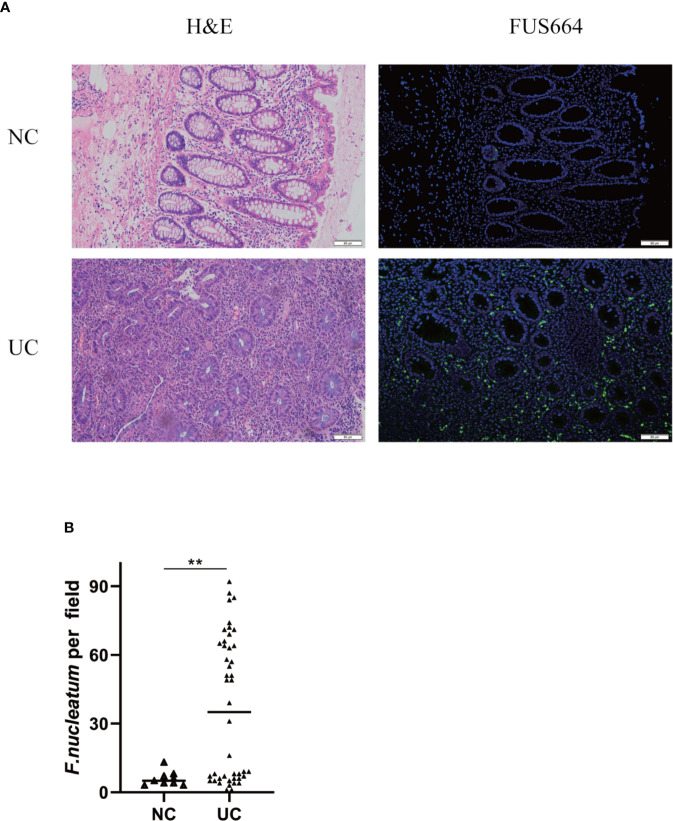
*F. nucleatum* is associated with UC. **(A)** Representative FISH images to assess the amount of *F. nucleatum* in UC (n = 44) and healthy control tissues (n = 9). FUS664 (green) is a FITC-conjugated *F. nucleatum*-specific oligonucleotide probe. Magnification, 200×. **(B)** The number of *F. nucleatum* was quantified in panel (**P < 0.01).

To examine the potential relationship between *F. nucleatum* and UC, we examined *F. nucleatum* abundance in paraffin sections of 44 UC tissues from patients and 9 normal tissues using FISH. *F. nucleatum* was detected in a higher percentage of UC tissues (24/44, 54.5%) than that in normal tissues (1/9, 11.1%; *P* <0.01; **Figure 1**). We then analyzed the relationship between *F. nucleatum* abundance and different clinical characteristics as shown in **Table 1**. The amount of *F. nucleatum* was positively correlated with the clinical course and clinical activity (*P <*0.05). Thus, these data suggest that the abundance *F. nucleatum* is possibly associated with clinical characteristics of UC.

**Table 1:** Clinical features of *F. nucleatum-*negative vs. *F. nucleatum-*positive UC.

	*F. nucleatum-*negative(n = 20)	*F. nucleatum-*positive(n = 24)	*P* value^a^
Gender			
Male	11	17	0.352
Female	9	7	
Age			
≤40	7	10	0.760
>40	13	14	
Location			
Rectum	6	2	0.174
Left side	5	7	
Total colitis	9	15	
Clinical course			
Active	11	21	0.021*
Remission	9	3	
Clinical activity			
Mild	11	5	0.043*
Moderate	6	9	
Severe	3	10	
HB			
Decreased	10	14	0.762
TP			
Decreased	3	5	0.710
WLB			
Decreased	2	7	0.150
HDL			
Decreased	1	2	1.000

^a^Chi-square test, *P < 0.05.

UC, ulcerative colitis.

### *F. nucleatum* Promotes IEC Autophagy *In Vitro*

**Figure 2:**
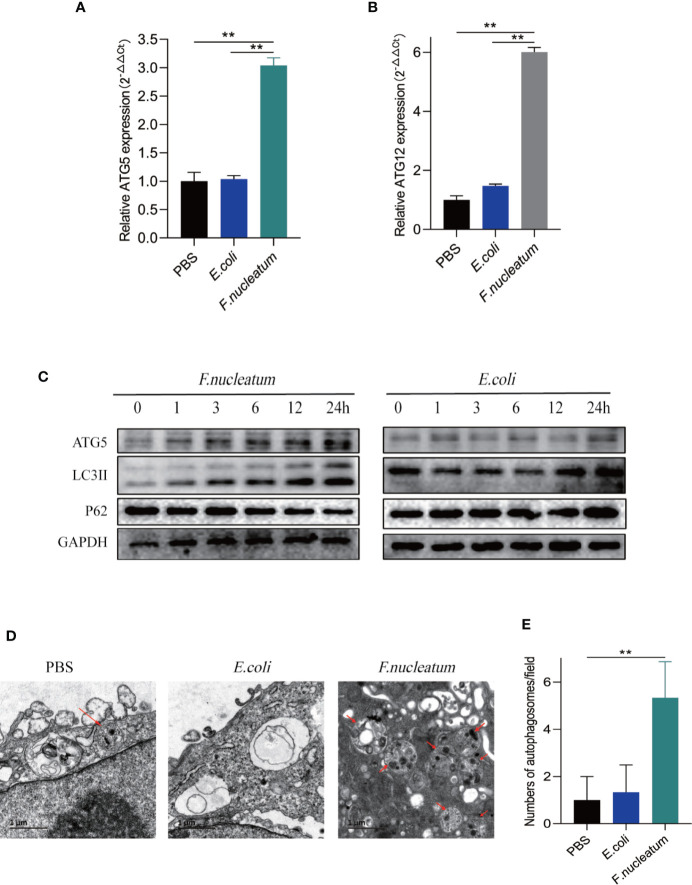
*F. nucleatum* promotes IEC autophagy pathway *in vitro*. **(A, B)** The mRNA expression of Atg5 and Atg12 was measured in NCM460 cells cocultured with PBS*, F. nucleatum* or *E. coli* for 24 h (*P < 0.05, **P < 0.01, and ***P < 0.001; unpaired Student’s t-test; the error bars indicate the SDs). **(C)** Western blot analysis was performed to measure the protein levels of Atg5, LC3 and P62 in NCM460 cells cocultured with *E. coli* or *F. nucleatum*. **(D)** Representative electron micrographs of autophagosomes (red arrows) in NCM460 cells infected with PBS, *E. coli* or *F. nucleatum*. Bar scale, 1 um. **(E)** Quantification of cells containing autophagosomes (from D) (**P < 0.01; unpaired Student’s t-test).

We hypothesized that *F. nucleatum* infection may aggravate intestinal inflammation by activating autophagy in UC. To test this hypothesis, we co-cultured NCM460 cells with *F. nucleatum*, *E. coli* (DH5α) or PBS (Control). Real-time PCR showed that *F. nucleatum* elevated autophagy-related gene ATG5 and ATG12 mRNA levels (**Figures 2A, B**). In line with this, Western blot results showed that *F. nucleatum* increased the expression of Atg5 and LC3II, and decreased the expression of P62 in a time-dependent manner (**Figure 2C**). These effects were not found in the NCM460 cells co-cultured with *E. coli*. These data suggest that *F. nucleatum* may drive autophagy activation in intestinal cells. Furthermore, Transmission electron microscopy confirmed the increase in the formation of autophagic vesicles in the *F. nucleatum* co-cultured NCM460 cells (**Figures 2D, E****)**. Collectively, our data indicate that *F. nucleatum* activates the autophagy pathway in IEC cells.

### *F. nucleatum* Promotes IEC Autophagy Activation *In Vivo*

**Figure 3:**
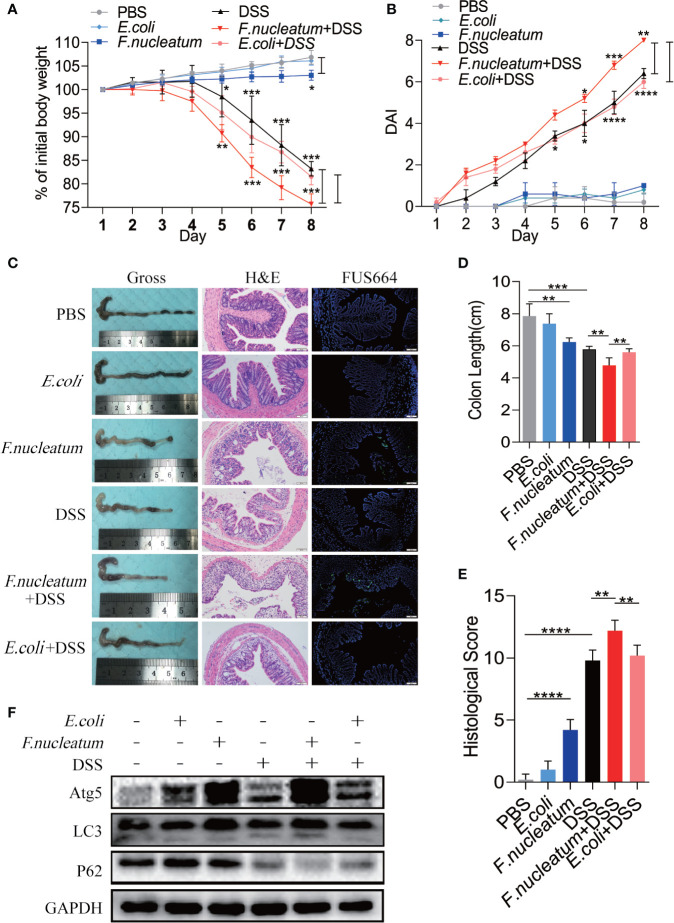
*F. nucleatum* promotes IEC Autophagy activation *in vivo* **(A, B)** Mice (n = 6 per group) were administered *F. nucleatum*, *E. coli* or PBS for 2 weeks and treated with 2.5% DSS for another 7 days. Colitis induction was evaluated by body weight loss **(A)** expressed as a percentage of the initial body weight and by the disease activity index (DAI) **(B)** (*P < 0.05, **P < 0.01, and ***P <0.001; one-way ANOVA combined with Bonferroni’s *post hoc* test. Error bars indicate SD). **(C**–**E)** Representative colon morphology and length of the mice described in **(A)** are shown in **(C)** and are quantified in **(D)**). Representative images of histological analyses are shown in **(C)** and are quantified in **(E)** (*P < 0.05, **P < 0.01, and ***P < 0.001; ****P < 0.0001; nonparametric Mann–Whitney test. Error bars indicate SD; 200 × magnification). **(F)** Western blot analysis was performed to measure the protein levels of Atg5, LC3 and P62 in the mouse tissues described in **(A)**.

To further understand the role of *F. nucleatum* in the development of UC, we established a DSS-induced experimental colitis model with *F. nucleatum* infection. We observed that mice co-treated with DSS and *F. nucleatum* showed more severe gut damage, including rapid weight loss (**Figure 3A**) and a higher disease activity index (DAI) (**Figure 3B**). Aggravation of the disease is accompanied by high abundance of *F. nucleatum* and *F. nucleatum* enhanced epithelial damage, including mucosal erosion, crypt loss, lymphocyte infiltration compared with *E. coli* (DH5α) (**Figure 3C**). The colon length of *F. nucleatum* + DSS group was significantly shorter than other groups (**Figure 3D**). In line with these results, a higher histological score (HS) was observed in *F. nucleatum* + DSS group than group treated with DSS, *F. nucleatum* or *E. coli* + DSS (**Figure 3E**). These data suggest that *F. nucleatum*, contrary to *E. coli* (DH5α), may exacerbate the histological features of DSS-induced colitis. Western blotting results showed higher levels of ATG5 and LC3II and a lower level of P62 in colitis tissues from *F. nucleatum* + DSS treated mice compared with that in the other groups (*F. nucleatum* group, DSS group and *E. coli* + DSS group) (**Figure 3F**), indicating that *F. nucleatum* specifically aggravates DSS-induced colitis through autophagy pathway. These results indicate that *F. nucleatum* may contribute to the development of UC *via* the autophagy pathway *in vivo*.

### *F. nucleatum* Leads to IEC Death In Vitro

**Figure 4:**
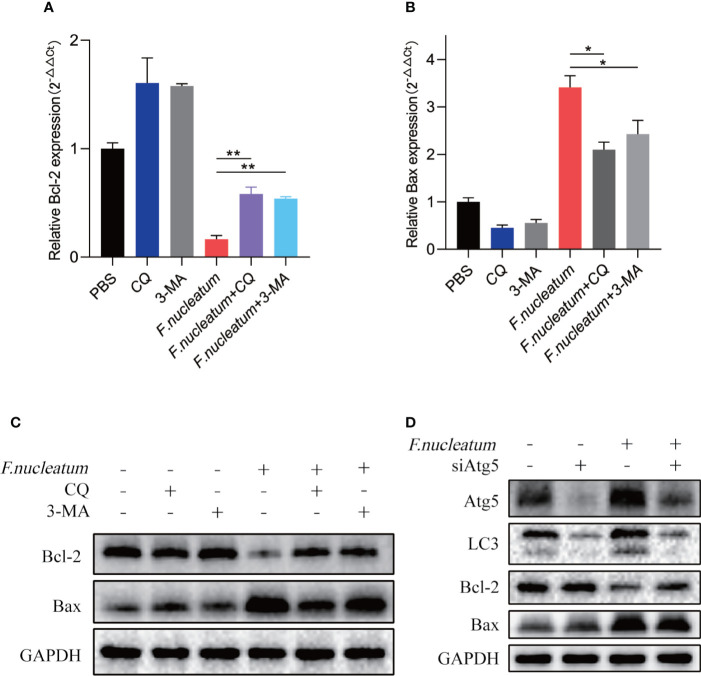
*F. nucleatum* promotes IEC death *in vitro*. **(A, B)** The mRNA expression of Bcl-2 and Bax was measured in NCM460 cells cocultured with PBS, CQ, 3-MA and *F. nucleatum* (*P < 0.05 and **P < 0.01; unpaired Student’s t-test; the error bars indicate the SDs). **(C)** Western blot analysis was performed to measure the protein levels of Bcl-2 and Bax in NCM460 cells cocultured with PBS, CQ, 3-MA and *F. nucleatum*. **(D)** Western blot analysis was performed to measure the protein levels of Atg5, LC3, Bcl-2 and Bax with NCM460 cells transfected with Atg5-targeting siRNA and cocultured with either PBS or *F. nucleatum* for 24 h.

Study found *F. nucleatum* could promote cell death in human gingival fibroblasts lymphocytes (Kaplan et al., 2010; Kang et al., 2019). To detect whether *F. nucleatum* promote cell death in IEC, we coculture NCM460 cells with *F. nucleatum*. RT-PCR and western bolt results showed that antiapoptotic protein Bcl-2 decreased in mRNA and protein levels, and proapoptotic protein Bax increased, indicating an increased level of apoptosis (**Figures 4A, B, C****)**. In addition, autophagy inhibitor of CQ and 3-MA or silencing Atg5 by siRNA knockdown reversed the level of *F. nucleatum*-induced apoptosis (**Figures 4C, D****)**, suggesting that *F. nucleatum* may promote apoptosis by activating autophagy.

### Chloroquine Deadens Excessive IEC Death in Colitis Mouse

**Figure 5:**
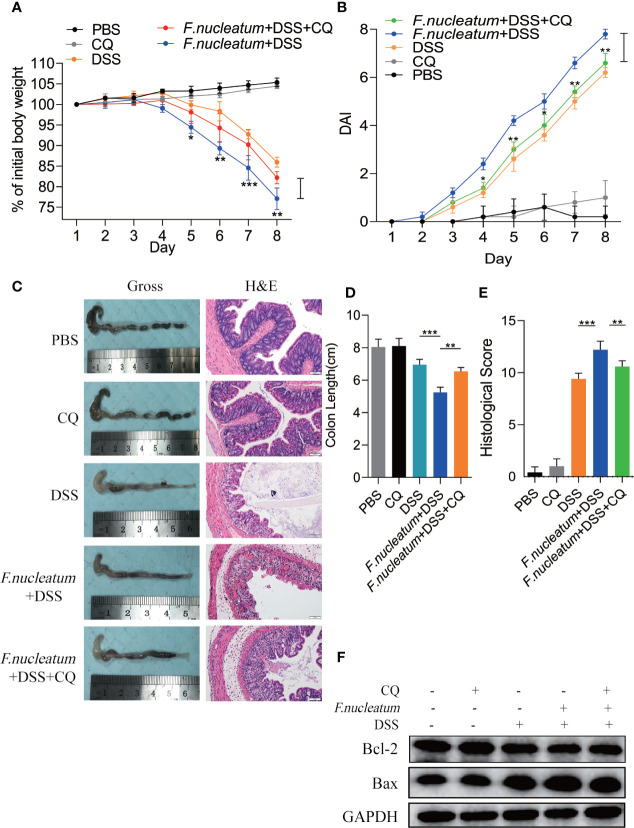
Inhibition of autophagy by CQ attenuates excessive cell death in the DSS-induced colitis mouse model *in vivo* and *in vitro* **(A, B)** For two weeks, Mice (n = 6 per group) were administered *F. nucleatum*, PBS and CQ was injected intraperitoneally every two days and then treated with 2.5% DSS for another 7 days. Colitis induction was evaluated by body weight loss **(A)** expressed as a percentage of the initial body weight and by the disease activity index (DAI) **(B)** (*P < 0.05, **P < 0.01, and ***P < 0.001; one-way ANOVA combined with Bonferroni’s *post hoc* test. Error bars indicate SD). **(C**–**E)** Representative colon morphology and length of the mice described in **(A)** are shown in **(C)** and are quantified in **(D)**. Representative images of histological analyses are shown in **(C)** and are quantified in **(E)** (*P < 0.05, **P < 0.01, and ***P < 0.001; one-way ANOVA combined with Bonferroni’s *post hoc* test. Error bars indicate SD; 200 × magnification). **(F)** Western blot analysis was performed to measure the protein levels of Bcl-2 and Bax in the mouse tissues described in **(A)**.

We found *F. nucleatum* promoted autophagy pathway in IECs *in vivo* and *in vitro*, therefore we want to know whether *F. nucleatum* will contribute to autophagic cell death to exacerbate the colitis. In our following experiments, CQ was used to inhibit lysosomal acidification, thus preventing autophagy by preventing the fusion and degradation of autophagosomes. We found that colitis mice treated with CQ showed a slower decline in body weight (**Figure 5A**), a lower DAI (**Figure 5B**), a longer colon length (**Figures 5C, D****)**, a lower HS (**Figure 5E**). HE results showed that CQ treatment attenuated inflammatory cell infiltration into mucosa and extensive damage of epithelium along with crypt destruction in *F. nucleatum* + DSS induced mice (**Figure 5C**). As a signaling pathway that regulates cell apoptosis and survival, increased level of Bax and decreased level of Bcl-2 has been studied as biomarkers of cell death. Western blot results showed that the use of CQ reversed the increased level of Bax and reduced level of Bcl-2 *in vivo* (**Figure 5F**). These results indicate that *F. nucleatum* may aggravate the development of IBD by promoting IEC autophagic death.

### LPS of *F. nucleatum* Promotes Autophagic Cell Death of IEC

**Figure 6:**
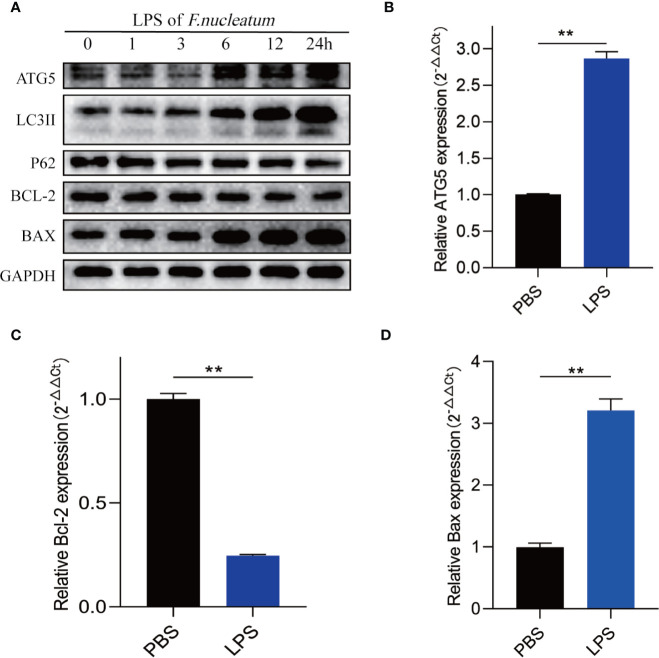
LPS of *F. nucleatum* promote autophagic cell death in UC. **(A)** Western blots showing that levels of ATG5, LC3II, Bax increase and expression of P62 and Bcl-2 decrease when NCM460 cells are challenged with LPS extracted from *F. nucleatum* over increasing time periods. **(B–D)** The mRNA expression of Atg5, Bcl-2 and Bax in NCM460 cells cocultured with LPS of *F. nucleatum* and PBS for 24 h (**P < 0.01; unpaired Student’s t-test; the error bars indicate the SDs).

To further understand how *F. nucleatum* affect the autophagy pathway, we extracted LPS from cultured *F. nucleatum* and treated NCM460 cells. Western blot results showed that LPS of *F. nucleatum* activated autophagy pathway in a time-dependent manner and it promotes the autophagic cell death of IEC (**Figure 6A**). Real-time PCR results showed that LPS also elevated ATG5 mRNA level and contributed to apoptosis level (**Figures 6B–D**). These findings indicate that *F. nucleatum* may contribute to autophagic cell death of IEC through its LPS.

## Discussion

Intestinal microbiota has been thought one of the major contributor in the pathogenesis of UC (Weingarden and Vaughn, 2017). Recent studies have found that *F. nucleatum*, a commensal within the oral cavity, is an emergent opportunistic pathogen of the gastrointestinal tract, having been abundantly found in patients suffering from UC (Nishida et al., 2018; Dinakaran et al., 2019). Here, we confirmed that *F. nucleatum* is an opportunistic risk factor of UC, which is closely related to the clinical characteristics of UC. In addition, we used DSS-induced colitis models to assess the effect of *F. nucleatum* in colitis. Through clinical specimen analysis and animal model verification, we have demonstrated that *F. nucleatum* is enriched in colorectal tissues of UC patients and exacerbates the severity of UC.

*F. nucleatum* have been confirmed to contribute to the CRC through promoting autophagy pathway and TNF-α expression in IEC (Yu et al., 2017). Infection with *F. nucleatum* was also found in UC patients (Zhou et al., 2016). However, the mechanisms of how *F. nucleatum* exacerbates colitis remains unclear. We examined the autophagy-associated protein (LC3 and p62) expression of IEC and colon tissues *in vivo* and *in vitro*. We found the presence of *F. nucleatum* was accompanied with elevated autophagy levels in IEC and colon tissues of DSS mice.

Next, we studied the mechanism by which *F. nucleatum* lead to IEC death. Elevated levels of apoptosis rate have been observed in the intestinal epithelium of patients with UC (Souza et al., 2004). Autophagy activation may be beneficial to cell survival by eliminating excess and damaged organelles, but excessive autophagy activation may cause autophagic cell death (Liu and Levine, 2015). We cocultured *E. coli* and *F. nucleatum* with intestinal epithelial cell lines, and found with *F. nucleatum* infection, the levels of autophagy and apoptosis were increased contrary to *E. coli*, but the use of CQ and 3-MA reduced the *F. nucleatum*-induce elevated apoptosis level of IEC and improved the integrity of mucosa. Therefore, we concluded that *F. nucleatum* may induce autophagic death of IEC to aggravate UC, and the effect can be mitigated by autophagy inhibitor CQ and 3-MA. One study found that calpain-mediated Atg5 cleavage provokes apoptosis cell death, which showed Atg5 could lead to cell death in an autophagy-independent manner (Yousefi et al., 2006). However, a previous study found that ATG5 can promote autophagy for cell death (Arakawa et al., 2017). Study in normal fibroblast cell lines also indicated that accumulation of autophagosomes and upregulation of ATG5 lead to cell death, which are attenuated by autophagy inhibition (Byun et al., 2009). Thus we examine the level of ATG5 in RNA and protein level, we found that when autophagic cell death occurs in IEC, the level of ATG5 increases, and silencing Atg5 could reduce the cell death and autophagy, confirming that ATG5 could induce IEC apoptosis in an autophagy-dependent manner.

As inhibitor of autophagy, CQ and 3-MA was widely-used in autophagy-related researches (Mauthe et al., 2018; Yang et al., 2019). Our study found that CQ and 3-MA inhibit *F. nucleatum*-induced autophagic cell death, CQ treatment attenuates the levels of the extent of inflammation, range of inflammation and crypt damage in *F. nucleatum*-treatment mice. In recent years, the role of autophagy regulators in IBD has been extensively studied. Therefore, our study finds that inhibiting autophagy by CQ and 3-MA may be used in the treatment of UC patient with high *F. nucleatum*, and this conclusion needs to be confirmed in clinical studies.

In addition, we investigated which component of *F. nucleatum* plays a role in mediating the autophagy of IEC. As the major component of the Gram-negative bacteria outer membrane, LPS is commonly used to establish intestinal injury models and induce inflammation with its antigenicity and cytotoxicity (Zhang et al., 2018; Xiong et al., 2019). Study found that LPS recognized by TLR4 to initiate a signaling cascade, of which NF-κB signaling pathway plays an vital role in inducing the inflammation and organ injury (Sun et al., 2015). LPS has been shown to induce intestinal epithelial cell autophagy, reduce intestinal cell proliferation, increase intestinal cell apoptosis, and ultimately lead to intestinal damage (Xie et al., 2019). Study have reported that *F. nucleatum*-related lipopolysaccharide (LPS) can induce macrophages to secrete TNF-α and promote inflammation (Grenier and Grignon, 2006), which both play an important role in pathogenesis of UC. Therefore, we chose to extract LPS to verify whether *F. nucleatum* exerts autophagy induction through its LPS. We demonstrated that LPS extracted from *F. nucleatum* can lead to autophagic death of IEC. However, we did not explore whether other components such as FadA play a role in autophagy activation.

From a clinical perspective, as the abundance of *F. nucleatum* is related with the severity of UC, treatment for autophagy may be a new target for UC patients with a high abundance of *F. nucleatum*. In addition, some conventional drugs used to treat UC have been shown to have autophagy regulating effect (Hooper et al., 2017), thus whether UC patients with *F. nucleatum* infection benefit from autophagy regulation of conventional treatment remains to be further elucidated.

Overall, our results demonstrate that *F. nucleatum* contribute to the development UC by inducing autophagic cell death of IEC. Moreover, the clinical study indicates that *F. nucleatum* is a risk factor for a high degree of disease activity in UC patients. Our research provides new evidence demonstrating the pathogenicity of *F. nucleatum* in UC and offer new sight to the treatment of intestinal microbe and autophagy regulation for the treatment of UC.

## Data Availability Statement

The original contributions presented in the study are included in the article/**Supplementary Material**. Further inquiries can be directed to the corresponding author.

## Ethics Statement

The studies involving human participants were reviewed and approved by Institutional Review Board of Renmin Hospital of Wuhan University, China. The patients/participants provided their written informed consent to participate in this study. The animal study was reviewed and approved by Institutional Review Board of Renmin Hospital of Wuhan University, China.

## Author Contributions

Study conception and design: WD, WS, YoC. Acquisition of clinical data: WS, YC, PC. Data analysis and interpretation and statistical analysis: WS, YoC, PC. Animal experiments: WS, YoC, PC, YC. Manuscript drafting: WS, YoC, WD. All authors contributed to the article and approved the submitted version.

## Funding

This work was supported by grants from the National Natural Science Foundation of China (No. 81870392, No. 81372551, and No. 81572426) and the Guiding Foundation of Renmin Hospital of Wuhan University (No. RMYD2018Z01).

## Conflict of Interest

The authors declare that the research was conducted in the absence of any commercial or financial relationships that could be construed as a potential conflict of interest.

## Supplementary Material

Supplementary Figure 1 *F. nucleatum* promotes IEC autophagy in vitro and in vivo. **(A–F)** The protein expression of Atg5, LC3 and P62 in NCM460 cells cocultured with F. nucleatum and E. coli were quantified. **(G–I)** The protein expression of Atg5, LC3 and P62 in mouse tissues were quantified. Data are expressed as mean ± SD for three independent experiments. Statistical significance is indicated as follows: *P < 0.05, **P < 0.01 and ***P < 0.001. **(J)** Western blotting was performed to measure the expression of Atg5 in NCM460 cells transfected with Atg5-targeting siRNA (siAtg5) and nontargeting siRNAs (NC).

Supplementary Figure 2 *F. nucleatum* and LPS promotes IEC autophagic cell death in vitro and in vivo. **(A, B)** The protein expression of Bcl-2 and Bax in NCM460 cells cocultured with PBS, CQ, 3-MA with or without F. nucleatum were quantified. **(C–F)** The protein expression of Atg5, LC3, Bcl-2 and Bax in NCM460 cells cocultured with PBS, F. nucleatum or siAtg5 were quantified. **(G, H)** The protein expression of Bcl-2 and Bax in colon tissues from mice were quantified. **(I–M)** The protein expression of Atg5, LC3, P62, Bcl-2 and Bax in NCM460 cells cocultured with LPS of F. nucleatum were quantified. Data are expressed as mean ± SD for three independent experiments. Statistical significance is indicated as follows: *P < 0.05, **P < 0.01, and ***P < 0.001.

Supplementary Figure 3 Low power field image corresponding to HE and FISH images of UC in **Figure 1**.

Supplementary Table 1 Clinical characteristics of *F. nucleatum*-negative vs. *F. nucleatum*-positive UC.
