# Supplementary figures and images for: Fusobacterium nucleatum Promotes the Development of Ulcerative Colitis by Inducing the Autophagic Cell Death of Intestinal Epithelial
Source: Front Cell Infect Microbiol. 2020 Nov 27;10:594806. doi: 10.3389/fcimb.2020.594806 (PMC7728699; doi:10.3389/fcimb.2020.594806)

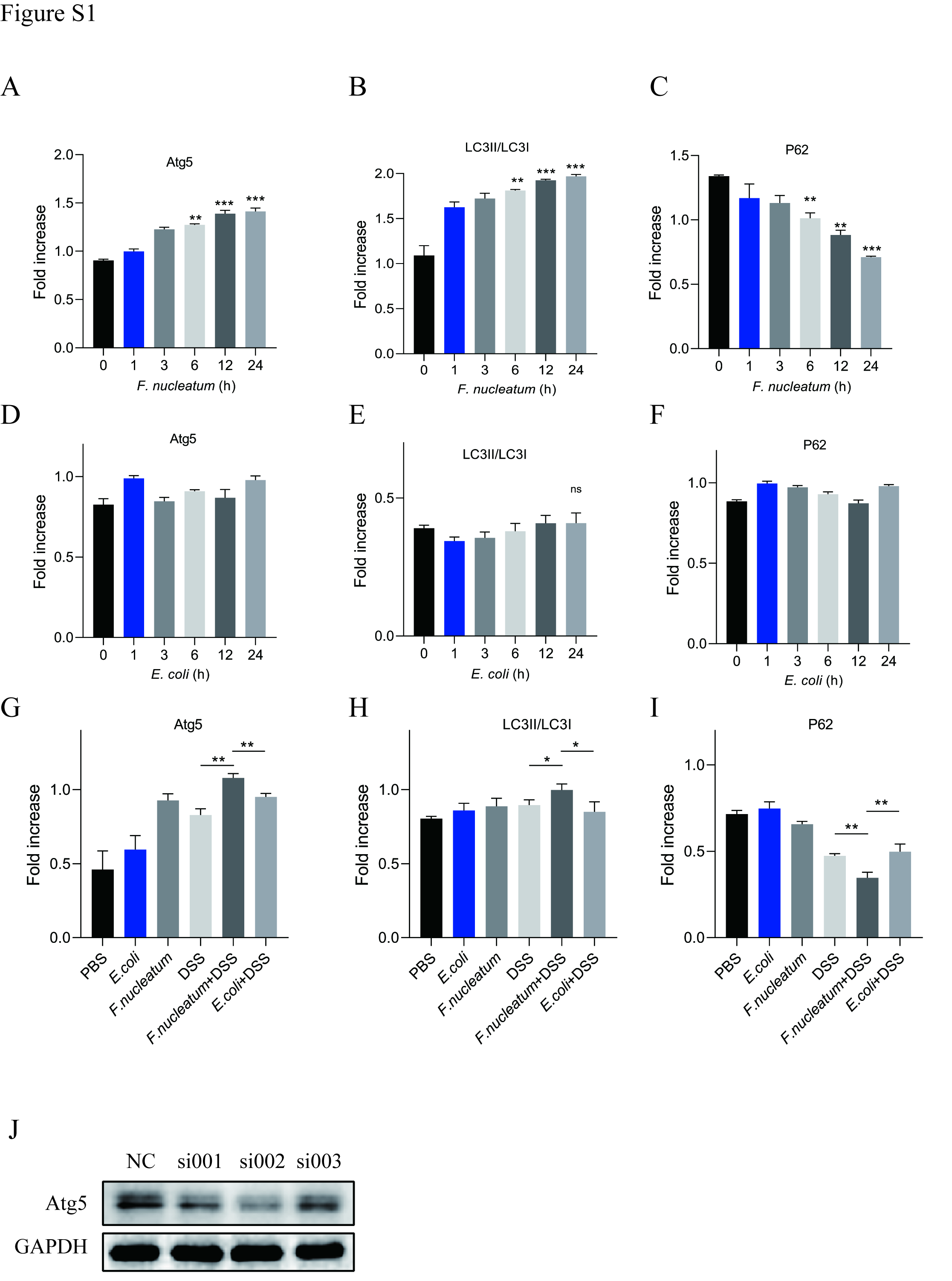

Supplement: Supplementary Figure 1 — F. nucleatum promotes IEC autophagy in vitro and in vivo. (A–F) The protein expression of Atg5, LC3 and P62 in NCM460 cells cocultured with F. nucleatum and E. coli were quantified. (G–I) The protein expression of Atg5, LC3 and P62 in mouse tissues were quantified. Data are expressed as mean ± SD for three independent experiments. Statistical significance is indicated as follows: *P < 0.05, **P < 0.01 and ***P < 0.001. (J) Western blotting was performed to measure the expression of Atg5 in NCM460 cells transfected with Atg5-targeting siRNA (siAtg5) and nontargeting siRNAs (NC). [file Image_1.tif]

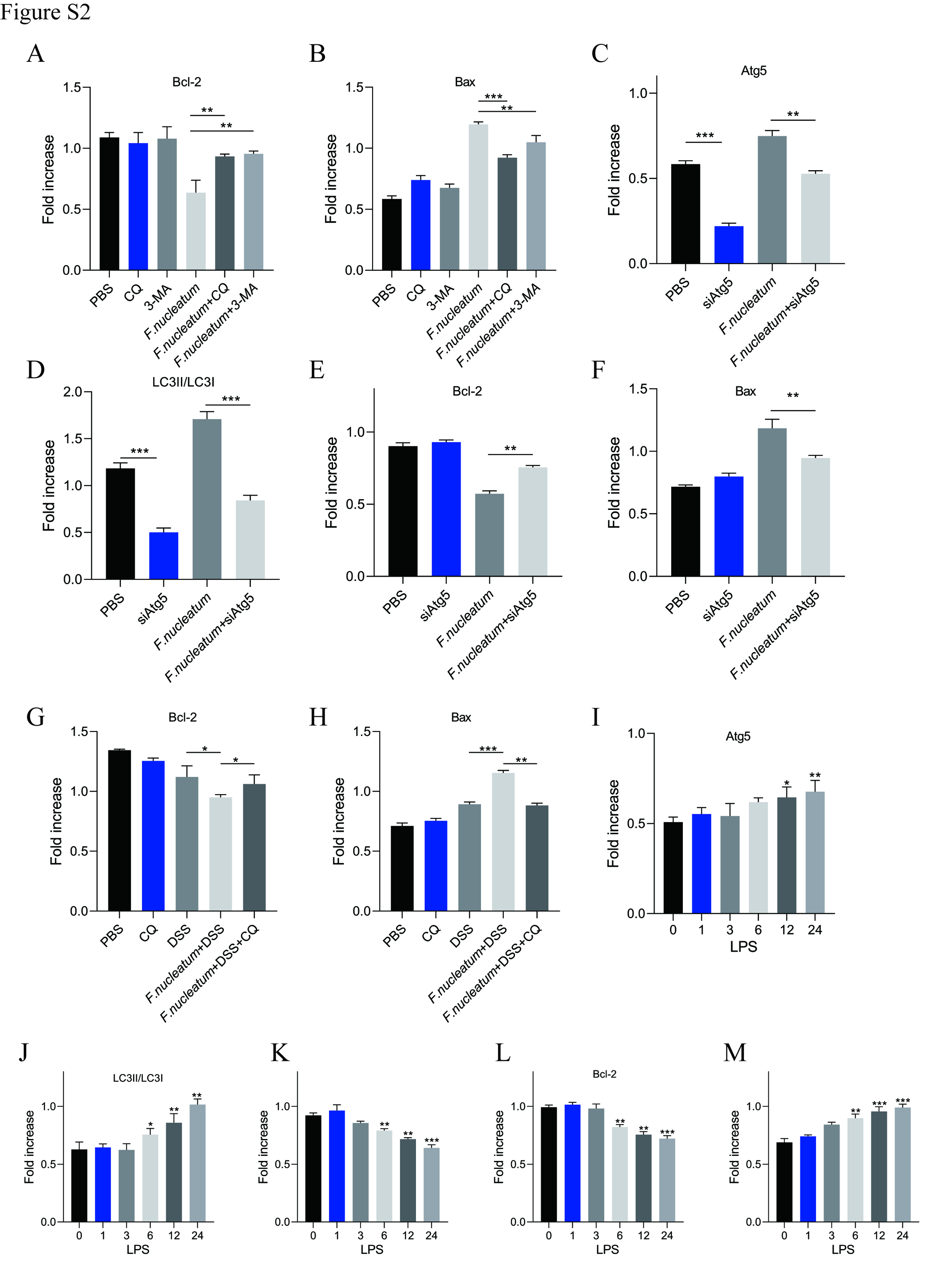

Supplement: Supplementary Figure 2 — F. nucleatum and LPS promotes IEC autophagic cell death in vitro and in vivo. (A, B) The protein expression of Bcl-2 and Bax in NCM460 cells cocultured with PBS, CQ, 3-MA with or without F. nucleatum were quantified. (C–F) The protein expression of Atg5, LC3, Bcl-2 and Bax in NCM460 cells cocultured with PBS, F. nucleatum or siAtg5 were quantified. (G, H) The protein expression of Bcl-2 and Bax in colon tissues from mice were quantified. (I–M) The protein expression of Atg5, LC3, P62, Bcl-2 and Bax in NCM460 cells cocultured with LPS of F. nucleatum were quantified. Data are expressed as mean ± SD for three independent experiments. Statistical significance is indicated as follows: *P < 0.05, **P < 0.01, and ***P < 0.001. [file Image_2.tif]

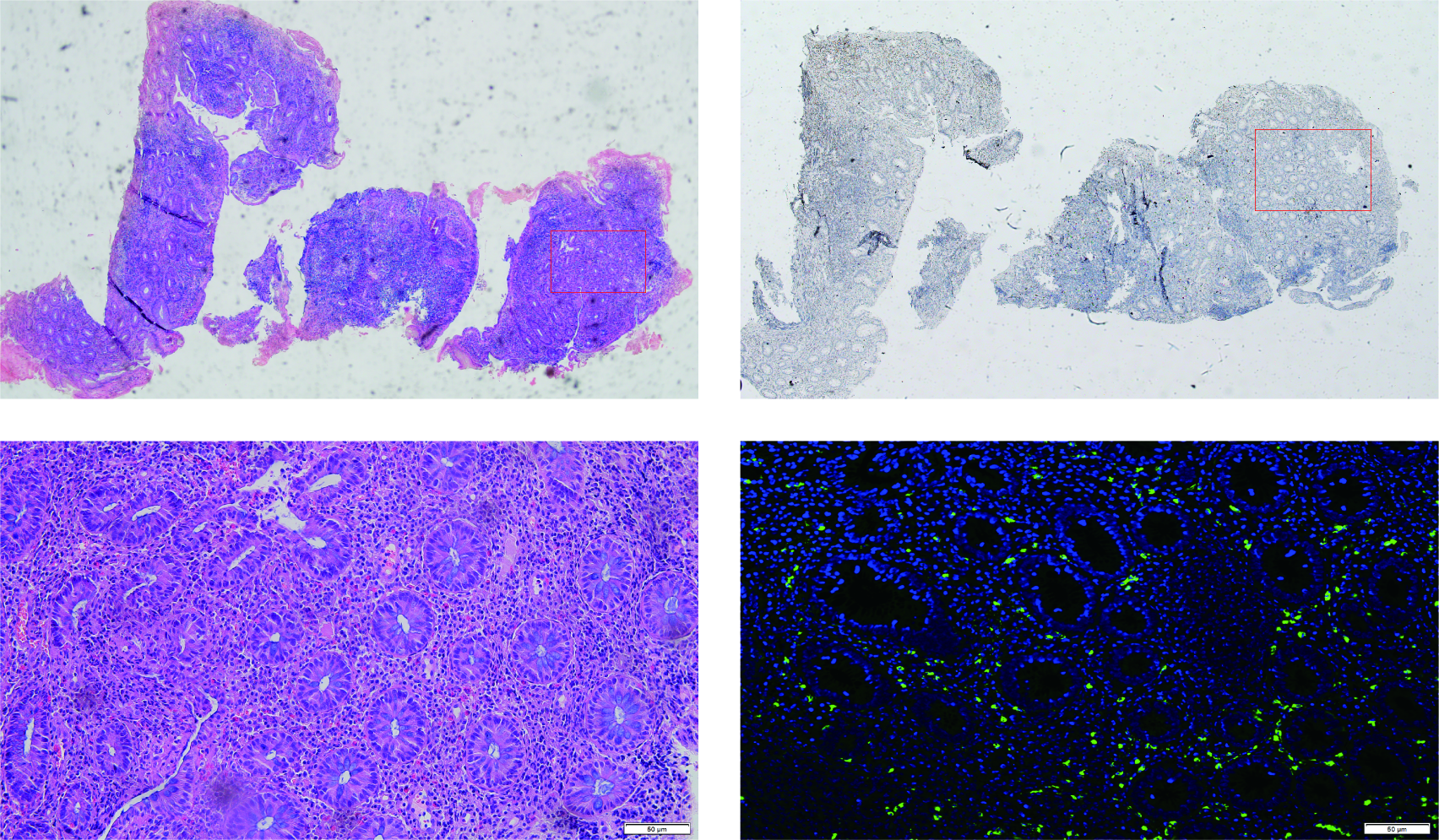

Supplement: Supplementary Figure 3 — Low power field image corresponding to HE and FISH images of UC in Figure 1. [file Image_3.tif]
