# Supplementary material for: Fusobacterium nucleatum Promotes the Development of Ulcerative Colitis by Inducing the Autophagic Cell Death of Intestinal Epithelial
Source: Front Cell Infect Microbiol. 2020 Nov 27;10:594806. doi: 10.3389/fcimb.2020.594806 (PMC7728699; doi:10.3389/fcimb.2020.594806)
Supplement: Supplementary Table 1 — Clinical characteristics of F. nucleatum-negative vs. F. nucleatum-positive UC. [file Table_1.docx]

Supplementary Table 1

| Primers |  | Sequence5’-3’ | Purpose |
| --- | --- | --- | --- |
| H-GAPDH | Forward | 5’- CA TCA TCCCTGCCTCTACTGG -3’ | qPCR |
|  | Reverse | 5’- GTGGGTGTCGCTGTTGAAGTC -3’ | qPCR |
| H-ATG5 | Forward | 5’- TGTTTA TTCGTCGGTTCA TTTTG -3’ | qPCR |
|  | Reverse | 5’- CAGCTTAGTGTTCCCTGCA TTC -3’ | qPCR |
| H-ATG12 | Forward | 5’- CTACTTCAA TTGCTGCTGGAGG -3’ | qPCR |
|  | Reverse | 5’- GCCAGCAGGTTCCTCTGTTC -3’ | qPCR |
| H-Bcl-2 | Forward | 5’- TGGGGTCATGTGTGTGGAGAG-3’ | qPCR |
|  | Reverse | 5’- AATCAAACAGAGGCCGCATG -3’ | qPCR |
| H-Bax | Forward | 5’- CCGAGAGGTCTTTTTCCGAG -3’ | qPCR |
|  | Reverse | 5’- AGGGACATCAGTCGCTTCAGT -3’ | qPCR |
